# Theoretical and Practical Issues That Are Relevant When Scaling Up hMSC Microcarrier Production Processes

**DOI:** 10.1155/2016/4760414

**Published:** 2016-02-11

**Authors:** Valentin Jossen, Cedric Schirmer, Dolman Mostafa Sindi, Regine Eibl, Matthias Kraume, Ralf Pörtner, Dieter Eibl

**Affiliations:** ^1^Institute of Chemistry and Biotechnology, Zurich University of Applied Sciences, Campus Grüental, 8820 Wädenswil, Switzerland; ^2^Department of Process Engineering, Chair of Chemical and Process Engineering, Technical University of Berlin, Strasse des 17.Juni 135, 10623 Berlin, Germany; ^3^Department of Bioprocess and Biosystems Engineering, Technical University of Hamburg, Denickestrasse 1, 21073 Hamburg, Germany

## Abstract

The potential of human mesenchymal stem cells (hMSCs) for allogeneic cell therapies has created a large amount of interest. However, this presupposes the availability of efficient scale-up procedures. Promising results have been reported for stirred bioreactors that operate with microcarriers. Recent publications focusing on microcarrier-based stirred bioreactors have demonstrated the successful use of Computational Fluid Dynamics (CFD) and suspension criteria (*N*_*S*1*u*_, *N*_*S*1_) for rapidly scaling up hMSC expansions from mL- to pilot scale. Nevertheless, one obstacle may be the formation of large microcarrier-cell-aggregates, which may result in mass transfer limitations and inhomogeneous distributions of stem cells in the culture broth. The dependence of microcarrier-cell-aggregate formation on impeller speed and shear stress levels was investigated for human adipose derived stromal/stem cells (hASCs) at the spinner scale by recording the Sauter mean diameter (*d*_32_) versus time. Cultivation at the suspension criteria provided *d*_32_ values between 0.2 and 0.7 mm, the highest cell densities (1.25 × 10^6^ cells mL^−1^ hASCs), and the highest expansion factors (117.0 ± 4.7 on day 7), while maintaining the expression of specific surface markers. Furthermore, suitability of the suspension criterion *N*_*S*1*u*_ was investigated for scaling up microcarrier-based processes in wave-mixed bioreactors for the first time.

## 1. Introduction

Cell-based therapies have become increasingly important in the field of regenerative medicine, as global revenues of approximately 1 billion US$ indicate [1, 2]. There has been an obvious growing interest in hMSCs, particularly in those that have shown great potential for a wide range of allogeneic therapies (e.g., dry eye-related macular degeneration, diabetes, Crohn's disease, graft versus host disease, and acute myocardial infarction [1, 3–8]). By September 2015, 171 Phase 1, 2, and 3 clinical trials with hMSCs had been run (https://www.clinicaltrials.gov/), a fact that comes as no surprise. Due to their existence in postnatal tissues (e.g., adipose tissue, bone marrow, umbilical tissue, blood, and peripheral blood) and lower regulatory restrictions than for embryonic stem cells, hMSCs are more easily accessible and more widely accepted for clinical applications [9–14]. The large amount of hMSCs required for one single therapeutic dose (35–350 million cells per dose) explains the demand for efficient and scalable* in vitro* expansion procedures [1, 15]. Although static stacked plate systems, with up to 40 layers, provide the desired cell numbers of up to 1 × 10^9^ cells in semicommercial and commercial production processes, it is difficult to ensure stem cell quantity and quality as the numbers of layers increase [16, 17].

Microcarrier-based bioreactors were identified as an alternative to planar cultivation technology that could meet the requirements in terms of production scale, bioprocess economics, and optimization [18]. The highest hMSC densities (1.4 × 10^5^–0.8 × 10^6^ cells mL^−1^) and maximum expansion factors (EFs) between 40 and 50 were achieved in stirred bioreactors operated with solid or porous microcarriers in a serum-supplemented (5–10% fetal bovine serum albumin, FBS) culture medium, cultivated for up to 21 days [19–30]. In order to successfully scale up microcarrier-based stirred bioreactor processes with hMSCs, Hewitt et al. [24] and Rafiq et al. [19] applied the suspension criterion *N*_*S*1_. This criterion that takes the high shear sensitivity [31, 32] of hMSCs into account can be attributed to Zwietering [33] and his studies from the late 1950s. *N*_*S*1_ represents the minimum impeller speed that just fully suspends the microcarriers at minimal shear stresses. However, it does not guarantee a homogenous dispersion of all microcarriers throughout a culture medium. Kaiser et al. [27] and Schirmaier et al. [20] introduced *N*_*S*1*u*_ criterion and proposed the antecedent prediction of the fluid flow pattern and hydrodynamic forces using Computational Fluid Dynamics (CFD) and Particle Image Velocimetry (PIV). *N*_*S*1*u*_ criterion represents the lower limit of *N*_*S*1_ criterion and allows for local movement of the microcarriers along the bioreactor bottom, but with none of the microcarriers at rest. By using *N*_*S*1*u*_ criterion, Schirmaier et al. [20] have achieved the highest number (1 × 10^10^) of both hASCs and EFs (41.7 within 7 days) in microcarrier-based stirred bioreactors at the pilot scale (35 L working volume) to date. However, as shown by Ferrari et al. [34], with bone marrow-derived hMSCs grown in spinner flasks on dextran microcarriers (Cytodex 1), large microcarrier-cell-aggregates can appear, which may result in mass transfer limitations and, finally, loss of stem cell properties, reduced cell growth, and even cell death. This raises the question of whether there is a dependence between microcarrier-cell-aggregate size, impeller speed, shear stress, cell quantity, and cell quality.

For this reason, one aim of our study was to investigate time-dependent hASC growth in spinners at different impeller speeds (taking the suspension criteria into account) and shear stress levels, while also taking the microcarrier-cell-aggregate size into account. All these investigations are based on the previously published characterization investigations (suspension studies, CFD simulations, and PIV measurements) of our group (Kaiser et al. [27]). The second aim was to examine whether it is possible to use *N*_*S*1*u*_ criterion for hMSC mass production processes in wave-mixed bioreactors with one-dimensional motion. In this type of bioreactor, mass transfer is accomplished by a propagating wave, whose intensity can be regulated by the bioreactor's rocking angle, rocking rate, and filling level. The wave is induced by rocking a fixed, surface-aerated bag [35–38] containing the medium and microcarriers to which the cells attach. Although this bioreactor type is well established in seed inoculum and microcarrier-based vaccine production processes with continuous animal cell lines, there are only two publications that describe its applicability to the expansion of hMSCs [39, 40]. Timmins et al. [39] cultivated human placental MSCs on CultiSpher-S microcarriers and achieved EFs of up to 16.3 within 7 days under low O_2_ (5%) conditions. In normoxic conditions Akerström [40] grew nonspecified hMSCs on Cytodex 3 microcarriers over 18 days and harvested 20 × 10^6^ cells, corresponding to an EF of 6. We decided to work with a BIOSTAT CultiBag RM 2L (optical version) and to adopt the shear stress at *N*_*S*1*u*_ for hASCs in spinner flasks (4.9 × 10^−3^ to 0.18 N m^−2^), which required previous suspension, CFD, and PIV investigations of the cultivation system.

## 2. Materials and Methods

### 2.1. Bioengineering Characterizations of the BIOSTAT CultiBag RM 2L

#### 2.1.1. Suspension Studies

The suspension experiments were carried out with a specially developed medium from Lonza containing 5% FBS and two different types of polystyrene-based microcarriers (Pall, USA). Three different working volumes (0.5 L, 1.0 L, and 1.5 L) and microcarrier solid fractions ranging from 0.7 to 2.1% were tested. The polystyrene-based microcarriers consist of particles with densities between 1090 and 1150 kg m^−3^ (MC-1) and between 1022 and 1030 kg m^−3^ (MC-2) and diameters between 160 and 200 *μ*m and between 125 and 200 *μ*m, respectively. The resulting nominal growth surfaces per gram were approximately 515 cm^2^ and 360 cm^2^. MC-1 was applied to establish an initial multiregression model for the prediction of the suspension criteria (*N*_*S*1*u*_, *N*_*S*1_) in the wave-mixed system and has no significance for the further cultivation studies described in Section 2.2.

In order to better assess the bioreactor bottom, a transparent, rigid rocking platform made of acrylic glass was used. In addition, a mirror was placed below the rocking platform to improve optical accessibility of the bioreactor bottom. Suspension characteristics were investigated for different rocking angles between 4° and 10°. The rocking angle was kept constant and the rocking rate was increased stepwise up to a maximum of 35 rpm. *N*_*S*1_ criterion for the wave-mixed bioreactor was defined as the combination of rocking rate and rocking angle, where the microcarriers make contact with the reactor bottom for no longer than 1 s. Likewise, for the stirred bioreactors, *N*_*S*1*u*_ criterion was the lower limit of *N*_*S*1_.

#### 2.1.2. CFD

The fluid flow inside the BIOSTAT CultiBag RM 2L was modelled using the Fluent 15 finite volume solver (ANSYS, Inc., USA). Due to the motion of the liquid interface, the Volume-of-Fluid (VOF) approach was used for the simulations. For this purpose, a set of single momentum equations, based on the Reynolds-averaged Navier-Stokes (RANS) equations, were solved. The interface between the phases was tracked over time using a balance equation for the fractional volume, which can be described for the *q*th phase by the following:

(1) $$\begin{array}{c} \frac{\partial }{\partial t}\left(\;\alpha_{q}\rho_{q}\;\right)+\nabla \left(\;\alpha_{q}\rho_{q}{\vec{w}}_{q}\;\right)=0. \end{array}$$

The terms ${\vec{w}}_{q}$, *α*_*q*_, and *ρ*_*q*_ in (1) denote the fluid velocity vector, the fluid density, and the volume fraction of the *q*th phase. By assuming a shared velocity field among the phases, a single momentum balance equation (see (2)) was solved for the entire fluid domain:

(2) ∂∂tρw→+∇ρqw→w→=−∇p+∇μ∇w→+∇w→T+ρg→+F→,

where $\vec{F}$ defines all the volumetric forces except gravity. In (2), the viscosity and the density are weighted mean values:

(3) ρ=∑αqρq,μ=∑αqμq

whose phase volume fractions are computed based on the following constraint:

(4) $$\begin{array}{c} \overset{n}{\underset{q=1}{\sum }}\alpha_{q}=1. \end{array}$$

A good approximation for the rocker-type motion of the bioreactor was described by a harmonic oscillation function, where the deflection angle (*φ*) at the time point *t* can be predicted by the following equation:

(5) $$\begin{array}{c} \varphi_{t}=\varphi_{max}\cdot \sin\left(\;\overset{-}{\omega }t\;\right). \end{array}$$

This resulted in (6), which was entered into Fluent as a user-defined function and describes the movement of the bag as a solid body:

(6) $$\begin{array}{c} {\overset{-}{\omega }}_{t}=\overset{-}{\omega }\left(\;\varphi_{max}\cdot \frac{\pi }{180}\;\right)\cdot \cos\left(\;\overset{-}{\omega }\cdot t\;\right). \end{array}$$

Turbulent flow conditions were modelled using *k*-*ω SST* model, where a set of transport equations for the turbulent kinetic energy *k*, the turbulent dissipation rate *ε*, and the specific turbulent dissipation rate *ω* were solved. Detailed information of the model is provided in [41, 42]. The volume of the pillow-like bag was discretized into 1.5 × 10^6^ tetrahedral control volumes, guaranteeing acceptable computational accuracy and a tolerable computational turnaround time. The simulations were performed for three different working volumes (0.5 L, 1.0 L, and 1.5 L) by patching the corresponding liquid phase. Rocking rates (14–35 rpm) and rocking angles (4–10°) were selected based on the results of the suspension studies. The velocity-pressure coupling and the prediction of volume fractions were accomplished using the SIMPLE algorithm and geo-reconstruction method provided by Fluent. The time-step size was fixed at 0.005 s. Convergence was assumed when the residuals dropped below 10^−5^. However, the number of iterations per time-step was restricted to 100 in order to limit the central processing unit turnaround time.

#### 2.1.3. PIV

PIV measurements were performed to verify the CFD model. For this purpose, a FlowMaster PIV system (LaVision, Germany) in conjunction with a double-pulsed Nd:YAG laser that generated a 1 mm thick laser sheet was used (*λ* = 532 nm, litron lasers, UK). The fluid flow pattern in the BIOSTAT CultiBag RM 2L was captured at two different positions (from the side and from below). For the side recordings, a specially constructed bag with a piece of acrylic glass along the centreline was used. This enabled process images to be recorded at the edges of the bag along the vertical laser plane, which was located in the middle of the half bag. The recordings from below were carried out for the whole bag. For this purpose, again a transparent rocking platform in combination with a mirror below the platform was used in order to provide optical accessibility to the reactor bottom. The laser sheet was horizontally positioned 1 cm above the bag bottom. An Imager Pro X4 CCD camera (LaVision, Germany) with a resolution of 2048 × 2048 pixels was used to acquire images and was positioned at a 90° angle relative to the laser field for the side measurements and directly on the mirror for the bottom investigations. DaVis® 8.2 software (LaVision, Germany) was used to control the camera, the traverse system, the laser, image acquisition, and flow field prediction. In order to visualise the fluid flow pattern, rhodamine-coated fluorescent particles with a density of 1.19 kg/m^−3^ (LaVision) were added to the bag. A set of 800 double frame images per position were recorded in order to obtain statistically significant results, based on an interrogation window of 8 × 8 pixels with an overlap of 50%. The measurements were performed for three working volumes (0.5 L, 1.0 L, and 1.5 L), different rocking angles (6° and 10°), and rocking rates (15 rpm, 25 rpm, and 35 rpm). Phase-locked measurements were recorded by means of a photoelectric barrier focused on the edge of the rocking platform. For each experimental set, images were made during the harmonic oscillation at momentary deflection angles of 4° to 10°.

### 2.2. Cultivation Studies

#### 2.2.1. Cells, Microcarriers, and Medium

Cryopreserved hASCs (Lonza Cologne GmbH, Germany) obtained from a single informed and consenting donor (hASCs, third passage, population doubling level of 10) were used for all expansions taking place under serum-reduced (5% FBS) conditions in a specially developed medium (Lonza, USA) on polystyrene-based microcarriers (Pall, USA). The microcarriers (MC-2) used had densities ranging from 1090 to 1150 kg m^−3^, particle sizes between 125 and 212 *μ*m, and a mean surface area of approximately 360 cm^2^ g^−1^.

#### 2.2.2. Analytics

Off-line samples were taken daily to determine glucose, lactate, glutamine, and ammonium by biosensors (enzymatic) and ion selective electrodes in the BioProfile 100Plus (Nova Biomedical, USA). In addition, 4′,6-diamidino-2-phenylindole (DAPI) staining was performed and microcarrier-cell-aggregates were measured. The aggregate measurements were performed based on macroscopic (hand camera) and microscopic pictures, which were analyzed by a user-defined MATLAB (MathWorks, Inc., USA) script and DaVis® 8.2 software. The cell densities were measured in triplicate per sample using a NucleoCounter NC-200 (ChemoMetec, Denmark). All microcarrier-cell-aggregates contained in the spinner flasks and the 2 L bag were washed with TrypLE Select (Gibco by Life Technologies, USA) and incubated for 30 min at 37°C before the hASC harvest.

Flow cytometric investigations (MACSQuant device from Miltenyi Biotec, Germany) were always performed after cell harvesting with microcarrier-free, purified hASCs samples. The samples were stained with fluorochrome-conjugated anti-human CD14, CD20, CD34, CD45, CD73, CD90, and CD105 antibodies (MSC Phenotyping Kit, Miltenyi Biotec, Germany), which represent minimal surface markers recommended by the International Society for Cellular Therapy.

#### 2.2.3. Corning Spinner Flask Cultivations

In order to investigate the influence of different impeller speeds on cell growth and aggregate formation, six different impeller speeds (25 rpm, 43 rpm, *N*_*S*1*u*_ = 49 rpm, *N*_*S*1_ = 63 rpm, 90 rpm, and 120 rpm) were studied under low-serum conditions (5%) in spinner flask (Corning, USA) experiments for MC solid fractions of 0.01%. For each condition, two spinner flasks (100 mL culture volume) with mean microcarrier growth surfaces of 360 cm^2^ were inoculated with cryopreserved hASCs (3 × 10^3^ cells cm^−2^) and cultivated for 8 days at 37°C, 5% CO_2_, and 80% humidity (normoxic).

Before inoculation, the microcarrier suspensions were equilibrated for 1 h, as recommended by the vendor. After inoculation, a 4 h attachment phase was realized to support cell attachment before the impeller was switched on. On day 4 after inoculation, 50% of the growth medium was replaced with fresh, preheated medium. For this purpose, the MCs with the attached cells were allowed to settle, before 50% of the medium was replaced with fresh, preheated medium. Cell attachment and harvest procedures were developed and optimized by Schirmaier et al. [20].

#### 2.2.4. BIOSTAT CultiBag RM 2L Proof-of-Concept Cultivation

The solid fraction of the polystyrene-based microcarriers was adjusted to 1.43% (7'722 cm^2^) for the proof-of-concept cultivation in the BIOSTAT CultiBag RM 2L. Equilibration of the microcarriers and inoculation of the cells were performed in two 1 L shake flasks. For this purpose, the microcarrier suspension was incubated overnight at 37°C, 5% CO_2,_ and 80% humidity. Each of the shake flasks was inoculated with 6500 cells cm^−2^ of the microcarriers for the cryopreserved hASCs. To promote cell attachment, a 20 h static attachment phase was found to be most suitable after inoculation. Afterwards, a portion of the microcarrier suspension (mean growth surface = 360 cm^2^) was transferred into a spinner flask as a control. The hASCs in the spinner flasks were cultivated as described for the spinner experiments in Section 2.2.3 (impeller speed = 49 rpm *N*_*S*1*u*_). The remaining microcarrier suspension (mean growth surface = 7'362 cm^2^) was transferred into a BIOSTAT CultiBag RM 2L. Preheated medium was then added to achieve a total working volume of 1.5 L. To achieve similar shear stresses as in the spinner flask, the BIOSTAT CultiBag RM 2L was overfilled with 500 mL medium. The rocking angle and rocking rate were set based on the biochemical engineering investigations (*N*_*S*1*u*_ = 4° and 31 rpm; Section 3.2.1). The hASCs were cultivated for 9 days at 37°C, pH 7.3, and 0.05 vvm. On day 5 after inoculation, the rocker platform was switched off and the bag was hung up to allow the MCs with attached cells to settle down. After approximately 15 min, 50% of the culture medium could be replaced with negligible cell and microcarrier lost.

## 3. Results and Discussion

### 3.1. Corning Spinner Flask

**Figure 1:**
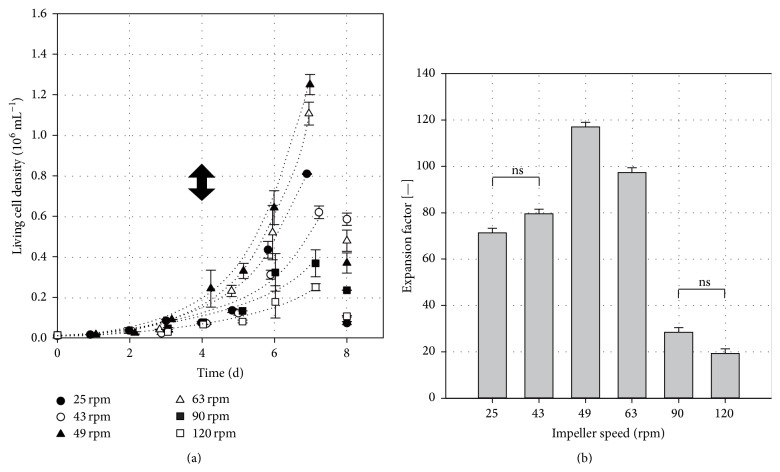
Results of hASC cultivations in the Corning spinner flasks. (a) Time-dependent profiles of living cell densities. The dotted lines represent the simulated growth characteristics of the hASCs in the exponential growth phase, based on the calculated specific growth rates. The black arrow indicates the 50% medium exchange on day 4. (b) Comparison of maximum expansion factors calculated for day 7. A one-way ANOVA (Holm-Sidak method, *n* = 2; *p* < 0.05) with pairwise comparison was performed for the maximum EFs. ns = not significant. The error bars represent the standard deviations of the EF given by the two cases of independent spinner cultivation per condition.

Figure 1(a) shows time-dependent profiles of living cell densities measured in the spinner flask runs. The impeller speed dependent growth parameters are summarized in Table 1. Maximum living cell densities between (0.25 ± 0.02) × 10^6^ hASCs mL^−1^ and (1.25 ± 0.05) × 10^6^ hASCs mL^−1^ were found 7 days after inoculation. The highest living cell densities were achieved at impeller speeds of 49 rpm and 63 rpm for *N*_*S*1*u*_ and *N*_*S*1_ criteria. Cell densities at the suspension criteria were four to five times higher than the living cell density at 120 rpm. This can be ascribed to the twofold to threefold lower local shear stress levels and the threefold to fivefold lower specific power inputs at *N*_*S*1*u*_ and *N*_*S*1_ (Table 1).

During the exponential growth phase, the fastest hASC growth (doubling time of 23.7 ± 0.1 h) was calculated for the spinner flask cultivation at 49 rpm (*N*_*S*1*u*_). The slowest hASC growth (doubling time of 41.3 ± 0.1 h) was found at 120 rpm. In spite of lower shear stresses, the hASCs also grew more slowly at impeller speeds below the suspension criteria. This might have been due to insufficient mixing and the resulting sedimentation of the microcarriers, since not all MCs were permanently suspended. Mass transfer limitations that impair cell growth can occur.

It can clearly be seen from Figure 1(b) that statistically significant, higher EFs (one-way ANOVA with pairwise comparison; Holm-Sidak method, *p* < 0.05, *n* = 2) were obtained for *N*_*S*1*u*_ (117 ± 4.7) and *N*_*S*1_ (97.4 ± 3.7) criteria. The lowest EFs {(28.5 ± 5.1) and (19.4 ± 1.3)} were achieved at the highest impeller speeds, which were up to four times lower than those at the lower impeller speeds of 25 rpm and 43 rpm.

The growth results (cell densities, doubling times, and EFs) support our hypothesis that operating a microcarrier-based stirred bioreactor at the lower suspension criterion *N*_*S*1*u*_ ensures maximum hMSC growth.

As is obvious from Table 2, the highest lactate production rate was determined at 120 rpm, while the maximum living cell density was the lowest. In contrast, the specific lactate production rate at 49 rpm (*N*_*S*1*u*_) was around 3.6 and 1.7 times lower compared to 120 rpm and 25 rpm. Furthermore, *Y*_lac/gluc_ indicates that the metabolization of glucose into energy is more efficient, when working at *N*_*S*1*u*_ criterion. After 7 days of cultivation, a decrease in the living cell density was observed, which was independent of the cultivation parameters. Because substrate and metabolite limitations can be excluded (concentrations at the end of the cultivation: glucose/glutamine ≥ 14.8/4.0 mmol L^−1^; lactate/ammonium ≤ 24.8/1.51 mmol L^−1^; [43, 44]), this might be due to the size of the microcarrier-cell-aggregates, which impair cell growth.

**Figure 2:**
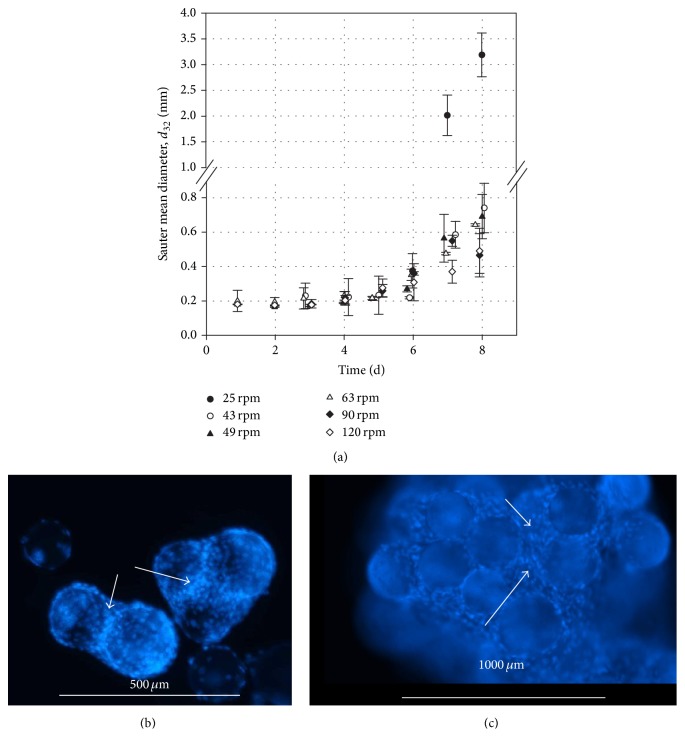
Microcarrier-cell-aggregate formation during the cultivation of the hASCs. (a) Time-dependent profiles of the Sauter mean diameters (*d*_32_) for all tested impeller speeds. (b) and (c) DAPI staining picture of microcarrier-cell-aggregate sample taken from a run at *N*_*S*1*u*_ suspension criterion (49 rpm, day 7). White arrow indicates that the cell growth takes place over the entire microcarrier surface and between the microcarriers. Focal plane was set in order to see the cell growth between the microcarriers.

Figure 2(a) clearly shows the microcarrier-cell-aggregate development. The time-dependent profiles of the Sauter mean diameters (*d*_32_) are shown for all tested impeller speeds. As expected, the highest Sauter mean diameter of up to 3.18 ± 0.42 mm was measured at the lowest impeller speed (25 rpm) on day 8 and was significantly higher compared to the other conditions. At this impeller speed, accumulation of larger aggregates below the impeller was observed. These findings are in good agreement with our previous CFD investigations published by Kaiser et al. [27]. Due to the circulation loop induced directly below the impeller, lower fluid velocities occur in this region, which promote the sedimentation of larger aggregates. The Sauter mean diameter at the lowest impeller speed was approximately seven times higher than at the suspension criteria. Interestingly, the Sauter mean diameter of 0.55 ± 0.06 mm on day 7 at 120 rpm was not significantly lower than those of the suspension criteria (*N*_*S*1*u*_: 0.58 ± 0.07 mm; *N*_*S*1_: 0.47 ± 0.03 mm), demonstrating that the threefold higher specific power input had no significant effect on the overall microcarrier-cell-aggregate size in the spinner flasks. The results indicate that the reduction in the living cell density might be ascribed to two main reasons: (I) too high local shear stresses (0.437 N m^−2^) which came along with higher lactate concentrations and (II) mass transfer limitations due to large microcarrier-cell-aggregate sizes (*d*_32_ > 0.6 mm) or too low impeller speeds (<49 rpm). Figures 2(b) and 2(c) illustrate DAPI staining picture of a microcarrier-cell-aggregate sample taken from a run (day 7) at *N*_*S*1*u*_ criterion.

At the end of the cultivation (days 7 and 8), the majority of the cells were observed between the microcarriers, especially in the bigger microcarrier-cell-aggregates (Figure 2(c)). A reduction of cell density prevails at Sauter mean diameters of approximately 0.6 mm, which was also noted at the end of the cultivation.

**Figure 3:**
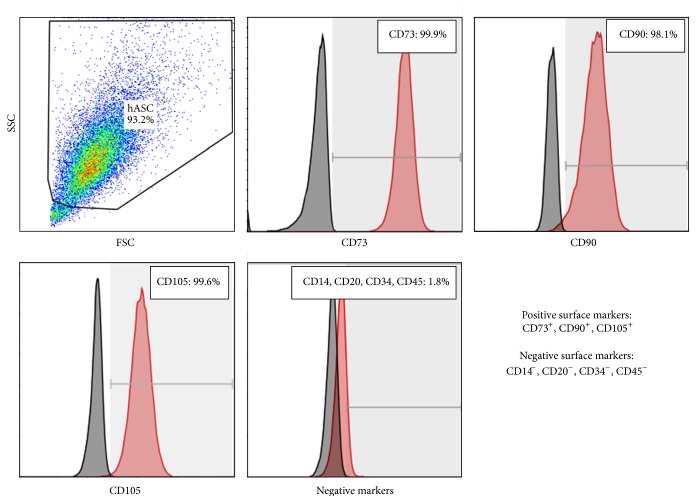
Results of flow cytometric analysis (FACS) of hASCs at the end of the cultivation at 49 rpm (day 7). The gates of the flow cytometric analysis were set based on isotype controls.

Any influence on the expression of specific surface markers (CD14, CD20, CD34, CD45, CD73, CD90, and CD105) was, however, not discovered, either at the maximum impeller speeds and resulting shear stress levels (maximum specific power inputs of 3.63 W m^−3^ and maximum local shear stress level of 0.437 N m^−2^) or at the maximum Sauter diameters (3.2 mm) that reflected the maximum microcarrier-cell-aggregation size. Because all flow cytometric results were in good agreement with each other, only those measured in samples from spinner runs at *N*_*S*1*u*_ are shown in Figure 3. The cells were highly positive (>95%) for CD73, CD90, and CD105 surface markers. The hematopoietic markers CD34 and CD45 as well as CD14 and CD20 were absent (<2%) from all samples.

### 3.2. BIOSTAT CultiBag RM 2L

#### 3.2.1. Suspension Characteristics

**Figure 4:**
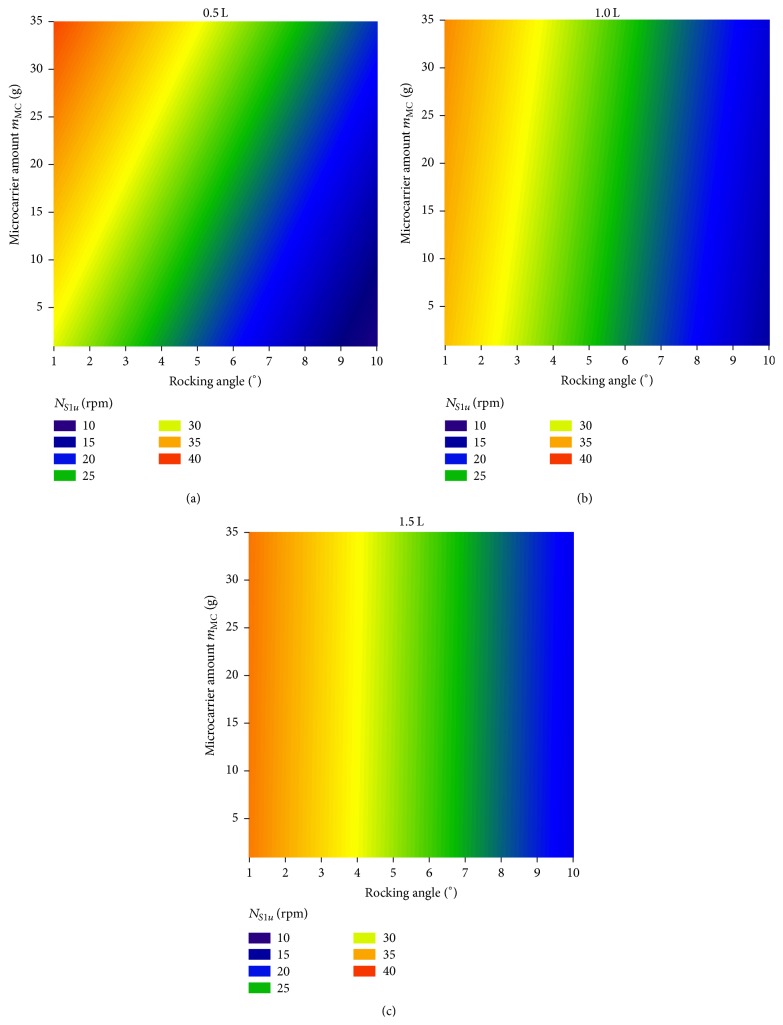
*N*_*S*1*u*_ criterion at the three different working volumes. The contour plots were created on the basis of the measured data and the multiple regression model.

In general, dune-like deposits of microcarriers were seen at rocking rates significantly below *N*_*S*1*u*_ criterion. This effect was independent of the rocking angle and can be explained by the oscillating fluid flow, in which a type of constructive and destructive interference takes place. As rocking rates increased the dune-like deposits decreased, due to the higher level of mixing. Almost complete suspension of the microcarriers was observed at *N*_*S*1*u*_ criterion. For all of the investigated conditions, *N*_*S*1_ criterion was fulfilled at 2.5–8.3% higher rocking rates (approximately 1 rpm). The results indicate that the difference between *N*_*S*1*u*_ and *N*_*S*1_ criteria in wave-mixed systems is much lower than in stirred bioreactors (20–40%) [21, 22, 27, 45, 46]. This phenomenon can be explained by the periodical deceleration and acceleration of the particles in wave-mixed bioreactors. The determined rocking rates that fulfil *N*_*S*1*u*_ criterion for rocking angles between 4 and 10° ranged between 12 and 26 rpm for a 0.5 L working volume, between 15 and 32 rpm for 1.0 L, and between 17 and 35 rpm for 1.5 L. The corresponding rocking rates for *N*_*S*1_ criterion were in a comparable range. Surprisingly, linear relationships were found between rocking rates and rocking angles for each specific microcarrier solid fraction. Based on a multiple regression analysis a correlation (see (7)) for *N*_*S*1*u*_ criterion was found:

(7) NS1u=−2.079·φmax+7.526·Vf−0.119·mMC+0.00537·AMC+0.0329·ρMC−6.039R=0.977,

where *φ*_max_ [°] defines the rocking angle, *V*_*f*_ [L] the working volume, *m*_MC_ [g] the amount of microcarriers, *A*_MC_ [cm^2^ 100 mL^−1^] the specific growth surface, and *ρ*_MC_ [kg m^−3^] the density of the microcarriers. The maximum deviation between the predicted and the measured values was approximately 3 rpm and, therefore, was acceptable. In Figure 4, *N*_*S*1*u*_ criterion is shown as a contour plot for the three different working volumes. As can be seen, the dependence of *N*_*S*1*u*_ criterion on the rocking angle decreases as the working volume rises.

#### 3.2.2. Fluid Flow

**Figure 5:**
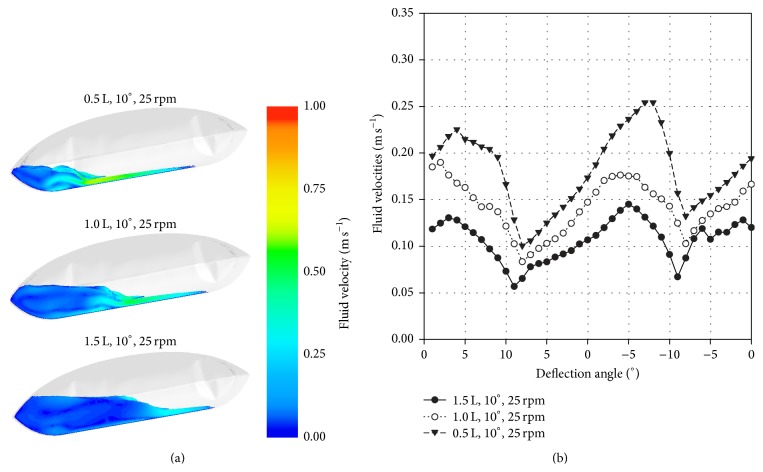
Comparison of fluid flow in the BIOSTAT CultiBag RM 2L at three different working volumes (0.5 L, 1.0 L, and 1.5 L). (a) Contour plots of CFD-predicted fluid flow patterns and fluid flow velocities for three different working volumes. The fluid flow patterns and the fluid flow velocities are shown for 25 rpm and 10°. (b) Comparison of volume-weighted mean fluid flow velocities over one period.

For a rocking rate of 25 rpm and a rocking angle of 10°, contour plots of the CFD-predicted fluid flow velocities along the mid bioreactor plane are shown in Figure 5(a). Significantly higher fluid flow velocities (0.75 m s^−1^) occur at maximum deflection for 0.5 L working volume. This was expected, since the motion of the free surface increases at lower working volumes. A clearly dampened fluid flow with fluid flow velocities of up to 0.55 m s^−1^ at maximum deflection was predicted for the higher working volume of 1.5 L. This trend can also be seen for the volume-weighted mean fluid flow velocities in Figure 5(b) and was independent of the rocking rate and the rocking angle. The maximum volume-weighted mean fluid flow velocities at 0.5 L, 25 rpm, and 10° were approximately 1.8 times higher than those at 1.5 L (0.14 m s^−1^). Furthermore, the simulation results indicated that a change in the rocking angle has a higher effect on the fluid flow velocities than a change in the rocking rate. The lowest volume-weighted mean fluid flow velocities of up to 0.05 m s^−1^ were obtained at 31 rpm and 4° and corresponded to *N*_*S*1*u*_ criterion in the BIOSTAT CultiBag RM 2L (1.43% microcarrier solid fraction). Kaiser et al. [27] predicted volume-weighted mean fluid flow velocities of up to 0.06 m s^−1^ for *N*_*S*1_ criterion in the spinner flask. Thus, working at 1.5 L is preferable for the expansion of the hASCs, when comparable flow conditions to those in spinner flasks are desired.

#### 3.2.3. Shear Stress and Specific Power Input

An overview of the shear stress distributions and specific power inputs present in the BIOSTAT CultiBag RM 2L (including working volume, rocking angle, and rocking rate) is summarized in Table 3. The shear stresses were calculated according to Wollny [47], where logarithmical normal distributions similar to those of stirred bioreactors were obtained [27, 48]. Unlike stirred bioreactors, the fluid flow behaviour in wave-mixed systems cannot be assumed to be constant. Hence, shear stress distributions were calculated for each deflection angle during a single bag oscillation.

**Figure 6:**
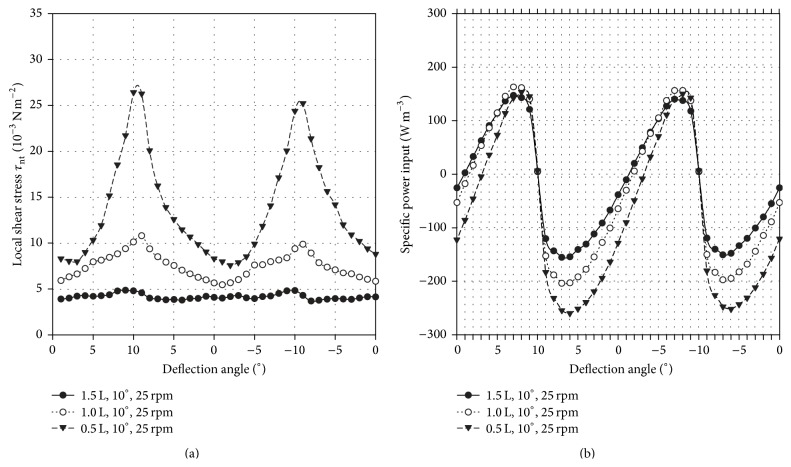
Angle-dependent profiles of volume-weighted mean shear stress levels (a) and specific power input (b) at 10° and 25 rpm and different working volumes.

In Figure 6(a), angle-dependent profiles of volume-weighted mean shear stresses are exemplarily presented for three different working volumes at 10° and 25 rpm. The local shear stresses follow a periodic oscillation, where the highest values occur at maximum deflection. This comes as no surprise, since the flowing fluid is decelerated to a lower velocity and then it accelerates in the other direction. The highest local shear stresses occurred at the lowest working volume of 0.5 L, with values that were up to 6 times higher than the local shear stresses for the 1.0 L and 1.5 L working volumes. The lowest local shear stress values (0.214 N m^−2^) were obtained at 1.5 L, 4°, and 31 rpm for *N*_*S*1_ criterion and a microcarrier solid volume fraction of 1.43%. These maximum local shear stresses are similar to those for *N*_*S*1_ criterion in the spinner flask. For 10° and 25 rpm, the angle-dependent specific power inputs based on the moment at the rotational axis are illustrated as an example in Figure 6(b). In general, the profiles of the specific power inputs correspond well to the local shear stresses. The highest specific power inputs appear shortly before the maximum deflection is reached. The highest specific power input of 262 W m^−3^ was determined at 0.5 L, whereas the lowest specific power input was 17.69 W m^−3^ and was achieved for 1.5 L, 4°, and 31 rpm (*N*_*S*1_ criterion). The power input results coincide with those obtained by Löffelholz et al. [36] and Eibl et al. [35].

#### 3.2.4. PIV Measurements

**Figure 7:**
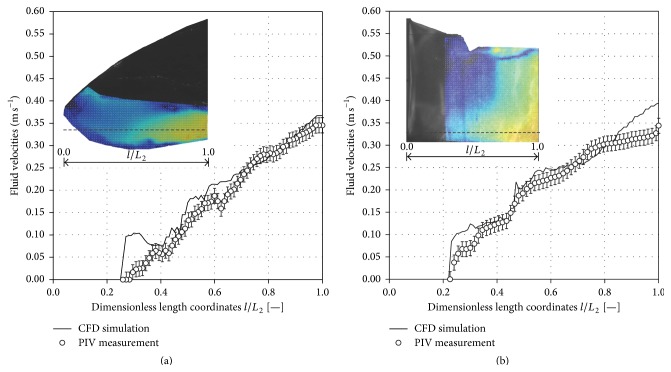
Comparison of PIV-measured (symbols) and CFD-predicted fluid flow velocities (solid line) through a horizontal line for side (a) and bottom view recordings (b). The comparison of the fluid flow velocities is given for operational parameters of 1.0 L, 25 rpm, and 10°. The error bars represent the standard deviation calculated over the 800 double frame images. The length coordinates were made dimensionless by the length of the field of view. The contour plots of the PIV data were scaled from 0.0 to 0.45 m s^−1^.

To validate the numerical models, a line was set along the measurement field after analysis of the PIV data (see Figure 7, contour plots of PIV data). For 25 rpm, 10°, and 1.0 L working volume, the CFD-predicted and PIV-measured fluid velocities are depicted as an example for the side (a) and the bottom view recordings (b) in Figure 7. The measurements were performed at a momentary deflection angle of 7° when lowering the bag. Only minor differences in the mean relative deviation (*δ*_*r*_), of less than 15%, were found between the predicted CFD and measured PIV data:

(8) $$\begin{array}{c} \delta_{r}=\frac{\sqrt{\left(1/X\right)\sum_{i=1}^{X}{\left(X_{exp_{i}}-X_{sim_{i}}\right)}^{2}}}{\sqrt{\left(1/X\right)\sum_{i=1}^{X}X_{{exp}_{i}}^{2}}}. \end{array}$$

The discrepancy between the data can be explained by the lowest deviations in the measuring angle and by the shape of the bag, since no fluid structure interactions were considered in the simulations. The largest deviations occur near the edges of the bag (see Figures 7(a) and 7(b);* l*/*L*_2_ 0.23–0.38). The dampening of the fluid flow at higher working volumes was also seen in the PIV measurements. Looking in addition at further results (e.g., spatial characteristics of the wave), it can be postulated that the established VOF model provides reliable fluid flow predictions.

#### 3.2.5. Proof-of-Concept Cultivation

**Figure 8:**
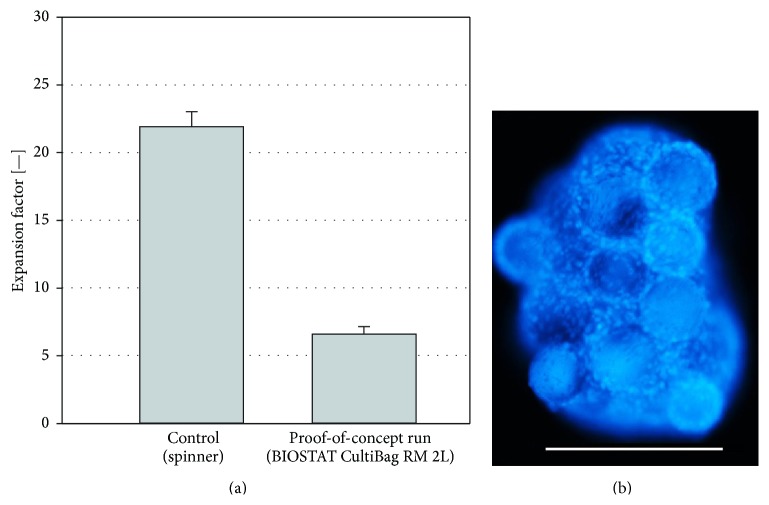
Results from the proof-of-concept cultivation in a BIOSTAT CultiBag RM 2L. (a) Comparison of the expansion factors in the BIOSTAT CultiBag RM 2L and the control spinner flask. The error bars represent the standard deviation. (b) DAPI staining picture of an aggregate at the end of the cultivation in the BIOSTAT CultiBag RM 2L. White bare = 500 *μ*m.

The proof-of-concept propagation of the hASCs in the BIOSTAT CultiBag RM 2L was successful, although the EF after 9 days was approximately three times lower (6.59 ± 0.56) than that in the control spinner (Figure 8(a)). The harvest provided 2.85 × 10^8^ hASCs. Akerström [40] reported a comparable EF, but over the double cultivation time. However, the cultivation process had not been optimized in terms of the attachment phase performed in shake flasks and the subsequent inoculation of the cells. The first microcarrier-cell-aggregates were already observed 3 days after inoculation. The diameters of the aggregates increased during the cultivation and reached a maximum size of approximately 6 mm (only a few aggregates) at the end of the cultivation.  Figure 8(b) shows representatively a DAPI-stained microcarrier-cell-aggregate sample at the end of the cultivation.

## 4. Summary and Conclusions

In this study, the superiority of the suspension criterion *N*_*S*1*u*_ for mass propagating hASCs in microcarrier-based stirred bioreactors was shown. The highest living cell densities and EFs of hASCs were achieved in stirred cultivation systems. These results confirm the observations of Kaiser et al. [27], Schirmaier et al. [20], and Jossen et al. [22].

In spinners, the lowest living cell densities were achieved at the maximum impeller speed (120 rpm). At this impeller speed, the maximum shear stresses were 130% higher than those at *N*_*S*1*u*_. Interestingly, the mean Sauter diameter, which was measured to evaluate the time-dependent microcarrier-cell-diameter, was lower than those at 25 rpm but comparable to those at the lower suspension criterion. Independent of the impeller speed, a decrease in the living cell densities was observed for mean Sauter diameters of 0.6 mm, even though shear stress levels were low and substrate limitation was excluded at the spinner scale. The reduction of the cell density might be a result of an undersupply of the cells in the centre of large microcarrier-cell-aggregates, although a change in the expression profile of the specific surface markers was not found. However, as reported for carrier-free cultivations with hASCs, cell aggregate diameters of 0.2 mm may already be critical and reduce the cell proliferation potential [34, 49]. Consequently, subsequent microcarrier-based productions of hASCs in spinners and stirred bioreactors not only should include extended cell quality control (differentiation and apoptosis assays) but also should pay attention to the number of cells in microcarrier-cell-aggregates. The question is whether a critical microcarrier-cell-aggregate size and number of cells in the aggregate can be defined and used as a harvest criterion for achieving maximum cell quantity with desired cell quality in hMSC expansions. These studies require subsequent investigations of diffusion limitations and examination of cell viability on the perimeter of the aggregates versus inside the aggregates.

Such findings represent one more step towards efficient and robust hASC mass production processes, which are also of interest for microcarrier-based, wave-mixed bioreactors, for which the suspension criteria were determined at the first time. An example of such a wave-mixed bioreactor is the BIOSTAT CultiBag RM 2L, which allowed 2.85 × 10^8^ hASCs to be harvested after a proof-of-concept cultivation performed at *N*_*S*1*u*_ conditions. Our established multiregression model makes the rapid definition of the suspension criteria for different working volumes possible and supports the optimization of microcarrier-based, wave-mixed bioreactors used for hASC cultivations. The consideration of further microcarrier types in the regression model would even allow hMSC expansions other than hASCs.

## Figures and Tables

**Table 1:** Summary of growth parameters, CFD-predicted specific power inputs (*P*/*V*), and local shear stress (LSS) levels.

*N* [rpm]	*P*/*V* [W m^−3^]	LSS^a^ [10^−3^ N m^−2^]	Total cell numbers on day 7 [10^7^ cells]	Living cell density on day 7 [10^6^ cells mL^−1^]	EFs [—]	*μ*_max_ and *t*_*d*_ [h^−1^] and [h]
25	0.21	3.21/69	8.1 ± 0.1	0.81 ± 0.01	71.4 ± 0.2	0.026 ± 0.001 26.7 ± 0.1
43	0.65	4.43/142	6.2 ± 0.6	0.62 ± 0.06	79.6 ± 3.2	0.022 ± 0.001 31.5 ± 0.2
49	0.80	4.96/187	12.5 ± 0.5	1.25 ± 0.05	117.0 ± 4.7	0.029 ± 0.001 23.7 ± 0.1
63	1.24	6.72/224	11.1 ± 0.6	1.11 ± 0.06	97.4 ± 3.7	0.028 ± 0.001 24.8 ± 0.2
90	2.24	10.22/325	3.7 ± 0.7	0.37 ± 0.07	28.5 ± 5.1	0.020 ± 0.002 34.4 ± 0.4
120	3.63	13.56/437	2.5 ± 0.2	0.25 ± 0.02	19.4 ± 1.3	0.017 ± 0.001 41.3 ± 0.1

^a^LSS; local shear stress given with volume-weighted mean/maximum values. LSS and *P*/*V* were adapted from Kaiser et al. [27].

**Table 2:** Specific metabolic consumption and production rates.

*N* [rpm]	−*q*_gluc_ [pmol cell^−1^ d^−1^]	*q*_lac_ [pmol cell^−1^ d^−1^]	*Y*_lac/gluc_ [mmol mmol^−1^]	*q*_NH_4_^+^_ [pmol cell^−1^ d^−1^]	*Y*_NH_4_^+^/gln_ [mmol mmol^−1^]
25	−1.86 ± 0.01	4.31 ± 0.03	2.87 ± 0.01	0.259 ± 0.004	2.65 ± 0.41
43	−2.30 ± 0.20	4.39 ± 0.30	1.94 ± 0.30	0.252 ± 0.003	2.66 ± 0.29
49	−1.58 ± 0.11	2.43 ± 0.17	1.55 ± 0.11	0.090 ± 0.001	2.46 ± 0.46
63	−1.08 ± 0.23	2.92 ± 1.04	2.51 ± 0.39	0.191 ± 0.052	2.36 ± 0.49
90	−4.09 ± 0.52	7.78 ± 1.39	1.89 ± 0.10	0.596 ± 0.133	2.43 ± 0.11
120	−5.07 ± 0.19	8.82 ± 0.11	1.74 ± 0.04	0.878 ± 0.085	2.57 ± 0.05

− *q*_gluc_: specific glucose consumption rate; *q*_lac_: specific lactate production rate; *q*_NH_4_^+^_: specific ammonia production rate; *Y*_lac/gluc_: specific lactate yield per unit glucose; *Y*_NH_4_^+^/gln_: specific ammonia yield per unit glutamine. (*n* = 2).

**Table 3:** Summary of predicted shear stress levels and specific power inputs in the BIOSTAT CultiBag RM 2L.

Working volume [L]	Rocking angle [°]	Rocking rate [rpm]	*P*/*V* ^a^ [W m^−3^]	MLSS^b^ [10^−3^ N m^−2^]
0.5	4	26	20.61/40.66	5.83/664
0.5	6	22	32.52/53.55	5.44/509
0.5	6	35	156.01/262.60	5.78/597
0.5	8	18	47.71/85.79	29.61/704
0.5	10	14	93.40/149.73	20.61/885
0.5	10	25	144.10/259.76	26.40/3162

1.0	4	29	34.40/65.33	4.91/916
1.0	6	25	53.70/98.16	4.75/1194
1.0	8	20	32.14/53.35	1.09/289
1.0	10	15	81.10/123.77	5.68/1012
1.0	10	25	118.32/203.76	10.80/4042

1.5	4	31	8.92/17.69	0.49/214
1.5	6	27	17.96/32.45	0.60/256
1.5	8	22	26.56/50.04	0.74/279
1.5	10	16	68.85/105.28	3.15/909
1.5	10	25	95.02/155.46	4.86/2959

^a^ *P*/*V*: mean and maximum values of specific power input. ^b^MLSS: maximum values of local volume-weighted mean and maximum shear stresses over one period.

## Abbreviations

ANOVA: Analysis of variance
CCD: Charged-coupled devices
CFD: Computational Fluid Dynamics
DAPI: 4′,6-Diamidino-2-phenylindole
EF: Expansion factor
FACS: Fluorescence-activated cell sorting
FBS: Fetal bovine serum
hASCs: Human adipose derived stromal/stem cells
hMSCs: Human mesenchymal stem cells
MSCs: Mesenchymal stem cells
PIV: Particle Image Velocimetry
RANS: Reynolds-averaged Navier-Stokes equations
SIMPLE: Semi-implicit method for pressure liked equations
VOF: Volume-of-Fluid
ns: Not significant.

## Symbols

### Latin Symbols

*A*_MC_: Specific growth surface of MCs [cm^2^ 100 mL]
*d*_32_: Sauter mean diameter [mm]
$\vec{F}$: Volumetric forces [N]
$\vec{g}$: Gravitational acceleration [9.81 m s^−2^]
*k*: Turbulent kinetic energy [m^2^ s^−2^]
*m*_MC_: Amount of MCs [g]
*N*_*S*1*u*_/*N*_*S*1_: Suspension criteria [rpm]
*p*: Pressure [Pa]
*P*/*V*: Specific power input [W m^−3^]
*t*: Time [s]
*t*_*d*_: Doubling time [h]
*V*_*f*_: Fluid volume [m^3^]
$\vec{w}$: Fluid velocity [m s^−1^].

### Greek Symbols

*α*: Phase volume fraction [—]
*ε*: Turbulent energy dissipation rate [m^2^ s^−3^]
*μ*: Laminar (molecular) viscosity [Pa s]
*π*: PI (3.14159) [—]
*ρ*: Density [kg m^−3^]
*μ*_max_: Maximum specific growth rate [h^−1^]
*φ*_*t*_: Deflection angle at time point *t* [°]
*φ*_max_: Maximum deflection angle [°]
*ω*: Specific turbulent dissipation rate [s^−1^]
$\overset{-}{\omega }$: Mean angular velocity [s^−1^].
